# Glucose Oxidase-Based Glucose-Sensitive Drug Delivery for Diabetes Treatment

**DOI:** 10.3390/polym9070255

**Published:** 2017-06-29

**Authors:** Li Zhao, Liyan Wang, Yuhan Zhang, Shanshan Xiao, Fei Bi, Jianyu Zhao, Guangqing Gai, Jianxun Ding

**Affiliations:** 1 Laboratory of Building Energy-Saving Technology Engineering, College of Material Science and Engineering, Jilin Jianzhu University, Changchun 130118, Jilin, China; zhaolizdl@163.com (L.Z.); wlynzy@163.com (L.W.); xiaoshanshan@jlju.edu.cn (S.X.); bifei1224@163.com (F.B.); 2 Department of Endocrinology, China-Japan Union Hospital of Jilin University, Changchun 130033, Jilin, China; 3 School of Phermaceuticel Science and Technology, Dalian University of Technology, Dalian 116024, Liaoning, China; yhzzhang@163.com; 4 Key Laboratory of Polymer Ecomaterials, Changchun Institute of Applied Chemistry, Chinese Academy of Sciences, Changchun 130022, Jilin, China

**Keywords:** glucose oxidase, glucose sensitivity, drug delivery, diabetes therapy

## Abstract

The glucose-sensitive drug delivery systems based on glucose oxidase (GOD), which exhibit highly promising applications in diabetes therapy, have attracted much more interest in recent years. The self-regulated drug delivery systems regulate drug release by glucose concentration automatically and continuously to control the blood glucose level (BGL) in normoglycemic state. This review covers the recent advances at the developments of GOD-based glucose-sensitive drug delivery systems and their in vivo applications for diabetes treatment. The applications of GOD-immobilized platforms, such as self-assembly layer-by-layer (LbL) films and polymer vesicles, cross-linking hydrogels and microgels, hybrid mesoporous silica nanoparticles, and microdevices fabricated with insulin reservoirs have been surveyed. The glucose-sensitive drug delivery systems based on GOD are expected to be a typical candidate for smart platforms for potential applications in diabetes therapy.

## 1. Introduction

Diabetes mellitus, one of the most serious health concerns following cancer and cardiovascular disease, is a metabolic disorder associated with abnormally elevated blood glucose level (BGL). The treatment of diabetes is urgent because the incidence of diabetics has been increasing sharply, which is predicted to be about 366 million all over the world in 2030 by the World Health Organization [1]. The frequently subcutaneous injection of exogenous insulin effectively controls the level of blood glucose. However, multiple subcutaneous insulin injections reduce patient compliance. Glucose-sensitive self-regulated drug delivery systems deliver drugs in direct response to the level of blood glucose with reduced injection time and an improved life quality of diabetics [2,3,4,5]. The smart drug delivery systems are expected to be a promising approach in diabetes therapy [6,7,8,9,10]. Glucose oxidase (GOD) as a sensing section is widely used in the glucose-sensitive drug delivery system. GOD is a homodimer composed of two identical 80 kDa subunits and two non-covalently bound flavin adenine dinucleotide complexes [11]. When GOD is incorporated with pH-responsive polymer materials, the enzymatic oxidation of glucose to gluconic acid catalyzed by GOD in glucose solution causes the pH change of microenvironment. Then the change of pH induces the swelling or shrinking of GOD-incorporated carriers or the acidic biodegradation of GOD-containing polymer matrices, resulting in the release of the preloaded drug [12,13,14]. In detail, the enzyme reactions are listed in Equations (1) and (2).

(1) $$D\mathrm{-glucose}+O_{2}\;\overset{\mathrm{GOD}}{\to }\;D\mathrm{-gluconolactone}+H_{2}O_{2}$$

(2) $$D\mathrm{-gluconolactone}+H_{2}O\;\to \;D\mathrm{-gluconic acid}$$

As shown in Equations (1) and (2), glucose is oxidized to gluconolactone by GOD firstly with the production of toxic intermediate hydrogen peroxide (H_2_O_2_), and then gluconolactone is rapidly hydrolyzed to gluconic acid in an aqueous environment. In the enzymatic oxidation, oxygen is consumed and H_2_O_2_ is produced when glucose is oxidized to gluconic acid. Therefore, in the GOD-based glucose-sensitive drug delivery, the produced H_2_O_2_ will prevent the reaction and inhibit the production of gluconic acid, which further inhibits the structure and property changes of pH-responsive polymer materials [15,16]. With a higher H_2_O_2_ concentration, the glucose-sensitivity of GOD-based drug carriers is limited. Measures are taken to assist the glucose sensitivity of GOD-mediated drug carriers. Some catalysts catalyze the decomposion of H_2_O_2_ to H_2_O, which can be co-immobilized with GOD on the matrix to reduce the concentration of H_2_O_2_ and maintain the glucose-sensitivity of GOD-incorporated matrices. When catalase (CAT) is combined with GOD and used in glucose-sensitive drug delivery, it not only decomposes H_2_O_2_ but also produces an oxygen molecule. The produced oxygen molecule further oxidizes glucose to gluconic acid catalyzed by GOD [17,18]. Furthermore, some other materials like hemoglobin (Hb) are usually used in the GOD-mediated matrices. The peroxidase activity of Hb catalyzes the reduction of H_2_O_2_ [19,20]. Besides, the introduction of H_2_O_2_- or hypoxia-sensitive groups in the materials is also designed for GOD-based glucose-sensitive drug delivery. Properties of platforms are changed due to the destruction of H_2_O_2_- or hypoxia-sensitive groups under the functions of produced H_2_O_2_ or hypoxia during the glucose oxidation catalyzed by GOD. As a return the payload is released, triggered by glucose.

**Figure 1:**
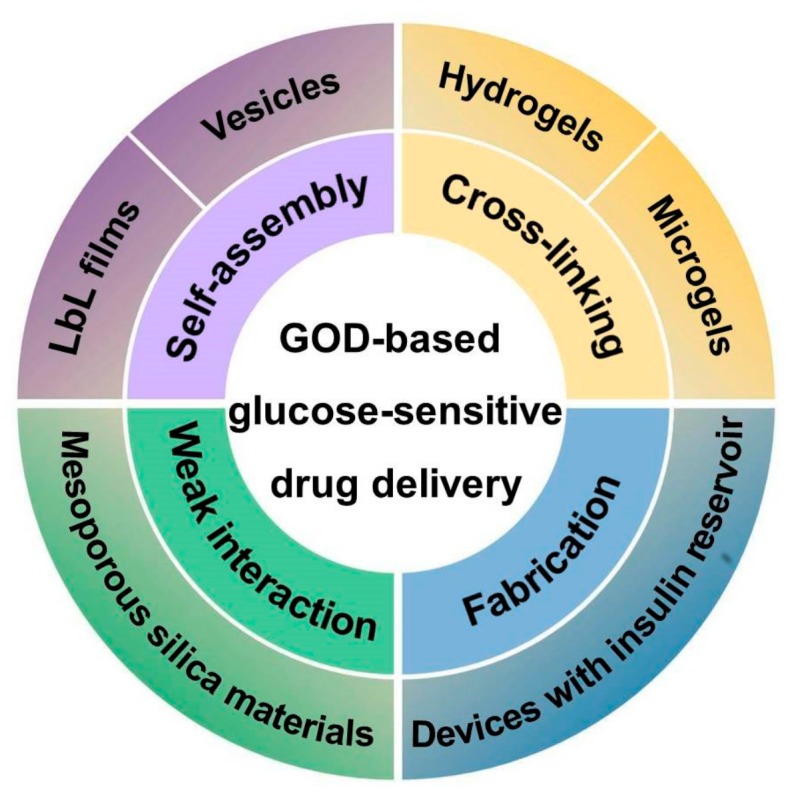
Glucose-sensitive self-regulated drug delivery platforms based on glucose oxidase (GOD).

GOD has been successfully used as a glucose-sensitive component integrated with pH-sensitive materials. GOD is incorporated in liposomes, and was usually modified to improve its hydrophobicity before its incorporation [21,22,23,24]. GOD-immobilized or incorporated membranes and microcapsules also have good glucose sensitivity with promising applications in self-regulated drug delivery [25,26,27]. Glucose-sensitive hydrogels based on GOD have attracted much more attention [28,29,30]. In recent years, glucose-sensitive drug delivery based on GOD has had great progress. In this article, the GOD-immobilized glucose-sensitive platforms with in vivo applications in self-regulated drug delivery with controlled regulation of blood glucose levels are reviewed. As shown in Figure 1, these GOD-based platforms include self-assembly films and polymer vesicles, cross-linking hydrogels and microgels, hybrid mesoporous silica nanoparticles, and microdevices fabricated with insulin reservoirs. The glucose-sensitive drug delivery mechanisms have been shown in Table 1. In summary, this article reviews the recent developments in glucose-sensitive platforms based on GOD.

## 2. GOD-Mediated Films and Vesicles

Self-assembly is an important branch of nanotechnology involving the organization of molecules or macromolecules into ordered structures by slack interactions [31]. All self-assembled structures are more stable thermodynamically than the single unassembled components. However, the external parameters easily influence the self-assembly systems. LbL films, microcapsules, and polymer vesicles and micelles can be fabricated by self-assembly technique with great applications in drug delivery, tissue engineering, biological detection, and so forth [32,33].

### 2.1. GOD-Mediated Films

The deposition protocol of layer-by-layer (LbL) self-assembly technique is based on the alternating adsorption of polymers with opposite charges on the surface of a solid substance through electrostatic attraction. The LbL method has been widely used for the development of functional microcapsules and films by the electrostatic deposition of polyelectrolytes, although LbL films are deposited through hydrogen bonding and biological affinity [34,35,36]. Stimuli-responsible microcapsules and films fabricated by LbL self-assembly technique have attracted particular interest due to their tailored membrane thickness and permeability. However, microcapsules immobilized with GOD for glucose-sensitive drug delivery have been reported on less since 2010, while the GOD-based films for glucose-sensitive controlled drug delivery have attracted growing scientific attention [20,37,38].

**Figure 2:**
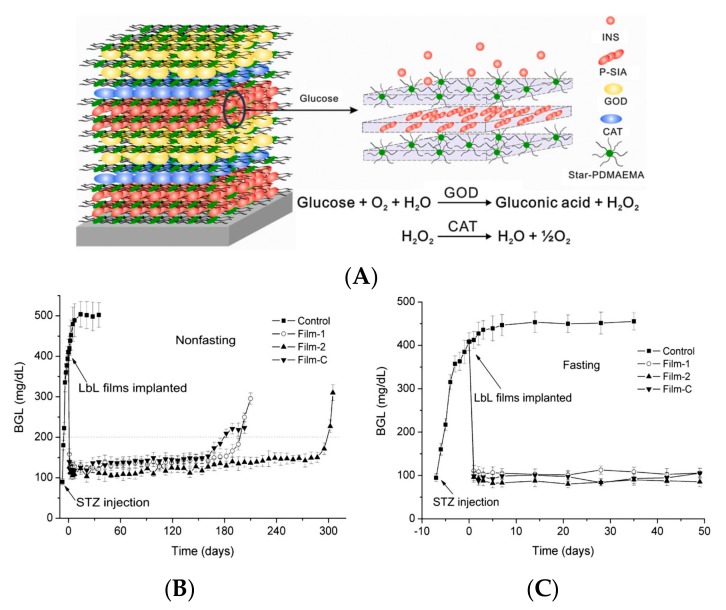
Glucose-sensitive insulin release mechanism and glycemic control in diabetic rats implanted with LbL films. (**A**) Schematic presentation of glucose-sensitive insulin release mechanism of LbL films under a coupled reaction of GOD and CAT; (**B**) BGL of diabetic rats (both nonfasting and fasting models) implanted with LbL films (*n* = 6); (**C**) Enlarged figure of (**B**). The control group is diabetic rats without treatment. The loading amounts of P-SIA in the films were Film-1 < Film-2, while Film-C was fabricated with denatured CAT to be the control sample of Film-2 to explore the role of CAT in this system in further experiments. (Reprinted with permission from [12]).

The LbL deposition, a bottom–up nanofabrication technique, has been used to construct insulin-containing nano- and micro-assemblies with entrapped GOD suitable for glucose-dependent insulin release systems. The GOD- and CAT-immobilized polytetrafluoroethylene (ePTFE)-*graft*-poly(acrylic acids) (PAAc) film (ePTFE-*g*-PAAc-*i*-GOD/CAT) was used to load insulin with a glucose-triggered permeation of insulin [39]. A glucose-sensitive multilayer film based on GOD and phenylboronic acid (PBA)-modified polyamidoamine dendrimer (PBA-PAMAM) were prepared by an alternate deposition through boronate ester bonds [40]. The boronate bond formed from the reaction between PBA and GOD, which is a glycoprotein containing a large amount of mannose residues. The multilayer film had pH-sensitive and glucose-induced decompositions consequently with insulin release. By LbL assembly method, a glucose-sensitive multilayer film was fabricated with a positive 21-arm star polymer, negative insulin and GOD [41]. The positively charged 21-arm poly(2-(dimethylamino)ethyl methacrylate) (star PDMAEMA) and negatively charged insulin were sequentially adsorbed on quartz slides, resulting in a four bilayers film. Then star PDMAEMA and GOD were sequentially adsorbed on the film of four bilayers, obtaining ((Star PDMAEMA/Insulin)_4_ + (Star PDMAEMA/GOD)_4_ + Star PDMAEMA). The release of insulin from the multilayer film was triggered by glucose, and the insulin release was shut off in the absence of glucose. In glucose solution, the gluconic acid decreased the pH of the microenvironment lower than the isoelectric point (5.4) of insulin and part of the insulin was changed to be positive. The insulin release was accelerated due to the electrostatic repulsion between star PDMAEMA and positively charged insulin. However, during insulin release, GOD was preserved in the films to maintain the structural integrity of the films. The reason was that the molecular weight of GOD was much higher than that of insulin and the isoelectric point (4.2) of GOD was lower than that of insulin (5.4). When the films were changed into solution without glucose, the pH of the microenvironment within the film increased and the insulin was negative. As a result, the electrostatic attraction between star PDMAEMA and insulin was enhanced, and then the release of insulin was decreased. Besides exploring the kinetics and mechanism of the glucose-regulated insulin release from the multilayer films, the authors studied the hypoglycemic effect of the films in vivo. In the treated group with insulin-filled films implanted, the plasma insulin concentration of diabetic rats was much higher, lasting for 14 days, while in the control group the plasma insulin concentration was trace. The released insulin in vivo had a key role to reduce BGL, which was controlled below 200 mg/dL for at least two weeks. This work offers a new route for developing self-regulated drug delivery systems. Also using star PDMAEMA, the same group fabricated multilayer films with star-PDMAEMA, GOD, CAT, and supramolecular assembly of porcine insulin (P-SIA) in the form of ((Star-PDMAEMA/P-SIA)_2_ + (Star-PDMAEMA/CAT)_1_ + (Star-PDMAEMA/GOD)_2_)_2_ + Star-PDMAEMA (Figure 2A) [12]. The multilayer films controlled the incorporated P-SIA release much longer, triggered by elevated glucose concentration. As shown in Figure 2B,C, the blood glucose concentrations of all the film-treated diabetic rats (both nonfasting and fasting models) were controlled in normal range (<200 mg/dL) for super long-term. The glycemic control of nonfasting and fasting diabetic rats were maintained for 175 and 49 days, respectively, indicating that there was no burst insulin release from the films. The insulin release profiles were confirmed by the plasma insulin levels and C-peptide measurements. The results are valuable to inspire more researchers to combine P-SIA with various functional drug delivery systems to promote the clinical applications of glucose-sensitive drug delivery systems.

### 2.2. GOD-Incorporated Vesicles

Polymers with opposite charges LbL self-assembled into films and microcapsules, while amphiphilic polymers self-assembled into polymer vesicles and micelles. Block copolymers form micelles from the more hydrophilic molecular variants and vesicles from amphiphiles of intermediate hydrophobicity [42]. However, the GOD-entrapped micelles cannot be obtained easily due to the high molecular weight of GOD [43]. Compared with GOD-entrapped micelles, GOD-incorporated vesicles have attracted more attention. GOD-incorporated vesicles for glucose-sensitive drug delivery systems must be combined with stimuli-response materials. The external stimuli induce the changes of materials and further change the structure of the polymer vesicles under enzymatic reactions accompanied by drug release.

**Figure 3:**
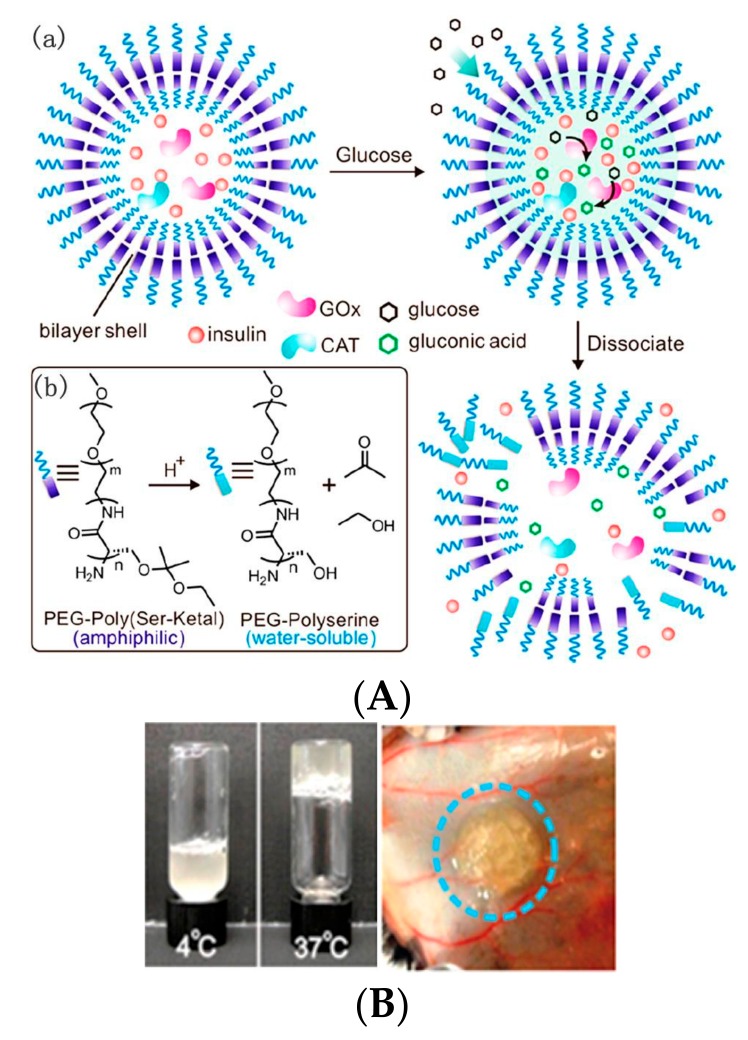
Injectable nanovesicle formulation made from enzyme-based glucose-responsive nanovesicle-embedded thermo-responsive matrix (**A**) Schematic of enzyme-based glucose-responsive nanovesicle and chemical structure of pH-sensitive copolymer PEG-poly(Ser-Ketal) (inset); (**B**) Immediate formation of hydrogel from nanovesicle integrated with thermo-responsive PF127 solution at 37 °C (left) in vitro and 3 min after subcutaneous injection (right). (Reprinted with permission from [44]).

One strategy is based on pH-sensitive polymers to design glucose-sensitive vesicles with immobilized GOD. Gluconic acid converted from glucose catalyzed by GOD causes the acidification of the microenvironment with a change to the vesicle structure and payload release. Gu and their coworkers have done extensive researches on GOD-immobilized polymer vesicles. Using pH-sensitive diblock polymers consisting of poly(ethylene glycol) (PEG) and ketal-modified polyserine (designated PEG-poly(Ser-Ketal)), the glucose-sensitive vesicles were prepared with recombinant human insulin, GOD, and CAT encapsulated [44]. Ketal is an acid-labile group. The gluconic acid produced from the glucose oxidization caused the acidic hydrolysis of ketals, resulting in the production of water soluble PEG-polyserine (Figure 3A). The water soluble PEG-polyserine led to the dissociation of the vesicles, followed by the insulin release. The vesicles were mixed with a thermo-responsive and biodegradable polymer Pluronic-127 (PF127) before subcutaneous injection to extend the long-term release intention. The mixture quickly formed a stable hydrogel, in which nanovesicles were evenly dispersed (Figure 3B). The nanovesicles were highly biocompatible and effective in regulating BGL in the normoglycemic range (<200 mg/dL for mouse) for up to five days.

GOD-based hypoxia-sensitive vesicles integrated with microneedle-array patches were firstly explored for regulation of insulin [45]. The hypoxia-sensitive vesicles, self-assembled from hypoxia-sensitive hyaluronic acid (HS-HA) conjugated with 2-nitroimidazole (NI), were dissociated in the hyperglycemic state. The reason was that hydrophobic NI was converted to hydrophilic 2-aminoimidazole through bioreduction under hypoxic condition during the enzymatic oxidation of glucose. The smart insulin patch effectively regulated the blood glucose for prolonged periods with an efficiently minimized risk of hypoglycemia.

**Figure 4:**
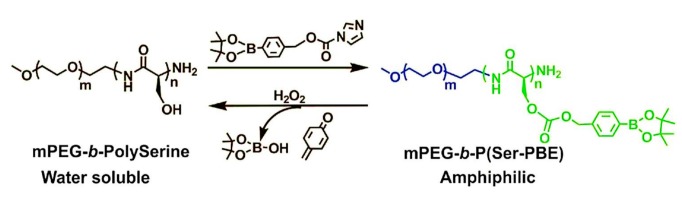
Schematic of chemical structure of mPEG-*b*-P(Ser-PBE) with H_2_O_2_-sensitive moieties and its degradation products. (Reprinted with permission from [47]).

Combining GOD with polymer incorporating H_2_O_2_-sensitive moieties, Napoli explored the GOD-encapsulated polymer vesicles from synthetic amphiphilic block copolymers consisting of poly(ethylene glycol) (PEG) and poly(propylene sulfide) (PPS) in form of PEG-PPS-PEG [46]. Thioether in the PPS block was converted into the more hydrophilic sulfoxides and sulfones upon exposure to an oxidative environment. The solubilization of polymer vesicles was increased due to the changing of the hydrophilic-lipophilic balance of amphiphilic block copolymers. Using the changes of the hydrophilicity of polymers, glucose-sensitive vesicles based on GOD had been designed. Gu group designed a novel kind of GOD-based H_2_O_2_-responsive vesicles with good glucose-sensitivity [47]. Block copolymers incorporated with PEG and phenylboronic ester (PBE)-conjugated polyserine (designated mPEG-*b*-P(Ser-PBE)) was used to assembly polymer vesicles with insulin and GOD encapsulated. The generated H_2_O_2_ during the enzymatic oxidation process degraded the pendant PBE, obtaining water soluble PEG-polyserine at physiological conditions (Figure 4). As a result, the structure changes of block copolymers made the dissociation of the vesicles followed by insulin release. To achieve excellent biocompatibility and sufficient stiffness, cross-linked hyaluronic acid (HA)-based microneedle-array patches were integrated with the polymer vesicles. The polymer vesicles demonstrated glucose-induced insulin release and maintained normoglycemia in diabetic mice. In addition, the applications of the H_2_O_2_-responsive artificial vesicles can be extended to deliver various therapeutics to treat other diseases.

**Figure 5:**
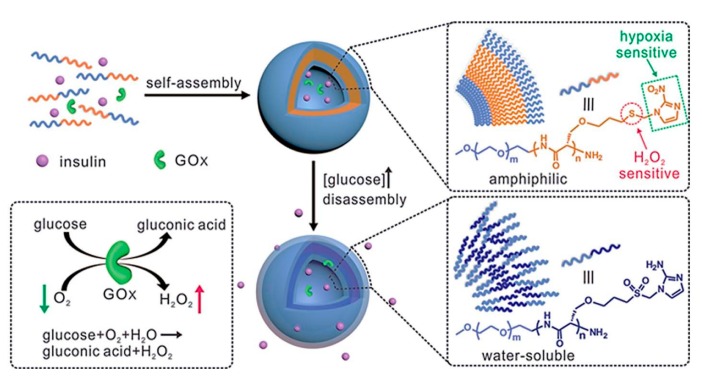
Schematic of formation and mechanism of hypoxia and H_2_O_2_ dual-sensitive polymersome-based vesicle comprised of PEG-poly(Ser-S-NI) for glucose-induced insulin delivery. (Reprinted with permission from [48]).

Another strategy to design glucose-sensitive drug delivery is combining GOD with hypoxia and H_2_O_2_ dual-sensitive polymer [48]. The hypoxia and H_2_O_2_ dual-sensitive copolymer, consisting of PEG and polyserine modified with 2-nitroimidazole via a thioether moiety (designated PEG-poly(Ser-S-NI)), self-assembled into bilayer vesicles with insulin and GOD encapsulation. H_2_O_2_ converted thioether into sulfone and hypoxia bioreducted NI into 2-aminoimidazole with enhanced hydrophilicity of the polymer, which resulted in the destruction of the vesicles and insulin release. These dual-sensitive vesicles integrated with a HA-based microneedle patch possessed high glucose-sensitive insulin release because of the synergistic reaction of hypoxia and H_2_O_2_ (Figure 5). The pulsatile insulin release regulated the BGL in diabetic mice effectively with minimal side effects. In addition, this dual-sensitive formulation strategy displays the potential benefit in controlled other therapeutic agents delivery under hypoxia and high oxidative stress.

Cationic and anionic polyelectrolytes self-assemble to films and amphiphilic block polymers self-assemble to polymer vesicles. With GOD immobilized or entrapped, glucose-sensitive LbL films and vesicles have been studied for self-regulated drug delivery with pulsatile release of insulin triggered by glucose. The membrane thickness and permeability of LbL self-assembly films are easily tailored with improved glucose-sensitivity. However, the mechanical strength of films is low and the drug loaded in the films easily leaks. With enhanced thickness and heightened electrostatic interaction, the mechanical strength of LbL films is improved. The drug-loading capacities of polymer vesicles are upregulated due to the larger inner volumes, but the unable structure depending on the preparation and application conditions limits their use in drug delivery systems. Integrated GOD-incorporated polymer vesicles with transcutaneous patches extend the stability and glucose-sensitivity of vesicles. Further efforts should focus on the enhanced stability of the platforms and activity of GOD to promote the clinical applications in diabetes therapy.

## 3. GOD-Mediated Hydrogels and Microgels

Even though LbL films with GOD immobilized or entrapped are proverbially used as glucose-sensitive drug delivery carriers, GOD-containing films suffer from low mechanical strength, resulting in unexpected drug leaking. While the polymer vesicles obtained by self-assembly of amphiphilic polymers provide larger inner volumes for drug loading, the unable structure limits their use in drug delivery systems. Compared with LbL films and vesicles, cross-linking pH-sensitive hydrogels and microgels provide suitable semiwet and three-dimensional (3D) environments for immobilizing or entrapping GOD generally. These platforms have the reversible swelling and shrinking changes with intact networks during the glucose-induced drug delivery, with promising applications in drug delivery.

### 3.1. GOD-Incorporated Hydrogels

Hydrogels are chemically or physically cross-linked polymer networks with 3D structures. Glucose-sensitive GOD-incorporated hydrogels are very suitable biomaterials for the development of self-regulated drug delivery systems due to their outstanding mechanical swelling or shrinking properties. Co-immobilization of GOD and CAT molecules into pH-sensitive hydrogels sense the change of glucose concentration and release the payload induced by glucose.

**Figure 6:**
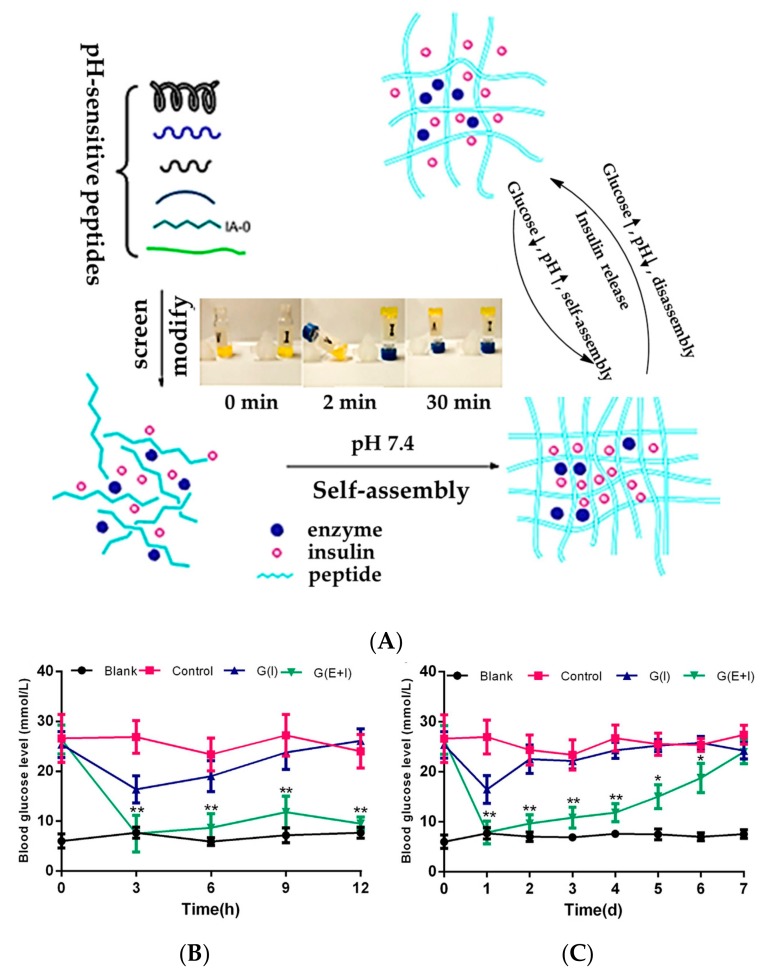
pH-sensitive self-assembled peptide hydrogels for glycemic control in diabetic mice. (**A**) Diagram of glucose-sensitive insulin delivery system using pH-sensitive self-assembled peptide hydrogel. The inset indicates the process of hydrogel formation; (**B**) BGL in the first 12 h and (**C**) in the long-term after administration of PBS solution (control), G(E+I) (hydrogel with both insulin and enzymes), and G(I) (hydrogel with insulin alone) to STZ-induced diabetic mice. The blank group was healthy mice. (Reprinted with permission from [52]).

The effect of immobilized GOD on diffusion and deformation of glucose-sensitive hydrogels was studied [49]. The simulations revealed that the optimization of GOD loading had the greatest impaction on the glucose sensitivity of hydrogels. In addition, the glucose sensitivity of the hydrogels was optimized by varying the thickness of the hydrogels’ strip [50]. The catalytic function of GOD encapsulated in alginate (MPA) hydrogels was confirmed [51]. Also using biodegradable materials, an injectable glucose-sensitive hydrogel using a pH-sensitive peptide hydrogel as a carrier was reported [52]. The hydrogel, quickly self-assembled by pH-sensitive peptide under physiological conditions, incorporates GOD and CAT with insulin loading (Figure 6A). The structure of peptide hydrogel was disassembled, which was induced by gluconic acid with a quick release of insulin. More importantly, the drug-loaded hydrogel had excellent in vivo hypoglycemic effects (Figure 6B). Hydrogel with both insulin and enzymes loaded (G(E+I)) have excellent in vivo hypoglycemic effects compared to that with insulin alone (G(I)) (Figure 6B). The BGL of diabetic mice treated by G(E+I) hydrogel was maintained below 11 mmol/L (normoglycemic state) for four days (Figure 6C). Most hydrogels with GOD incorporated are prepared with cross-linking structures using a small molecule as a cross-linker, while the pH-sensitive peptide hydrogel was obtained by self-assembly of peptide. The glucose-sensitivity relied on the significant repulsion of alkaline amino acid side chains due to the gluconic acid, which induced the unfolding of individual hairpins on peptide, disassembly of hydrogel and insulin release. This work provides important guidelines for the design of biocompatible and biodegradable glucose-sensitive platforms based on GOD.

**Figure 7:**
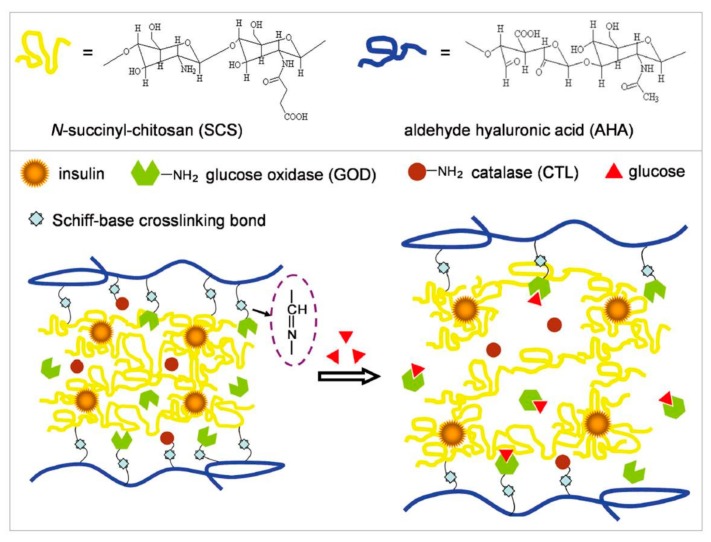
Schematic representation of structures of SCS and AHA, and SCS and AHA-based Schiff-base crosslinking hydrogel using for insulin release triggered by glucose. (Reprinted with permission from [54]).

Chitosan (CS) is another kind of biocompatible and degradable material with great applications in the design of drug delivery systems. The intelligent semi-interpenetrating network (semi-IPN) hydrogels were fabricated by free radical polymerization of CS, acrylamide (AAm), and polyethylene glycol (PEG) using *N*,*N*′-methylenebisacrylamide as a cross-linker [53]. Via a swelling-diffusion method, GOD and CAT were immobilized and insulin was loaded into the semi-IPN hydrogels. CAT assists the enzymatic reaction of GOD for the consummation of undesired H_2_O_2_ with regeneration of oxygen (O_2_), resulting in lasted insulin release. In addition, the studies of the insulin release kinetics by various models provided theoretical support for the hydrogels used for glucose-sensitive insulin-controlled release. Injectable in situ forming Schiff-base crosslinking hydrogels based on *N*-succinyl-chitosan (SCS) and aldehyde hyaluronic acid (AHA) was designed as shown in Figure 7 [54]. The gelation rate of composite hydrogels was related with the volume ratios of SCS/AHA, and all gelation occurred within 3 min with insulin and enzymes loaded. Insulin cumulative release behavior from the hydrogels in vitro induced by glucose was studied. The glucose-sensitive Schiff-base crosslinking hydrogel system immobilized with enzymes provides a new strategy for designing glucose-sensitive drug delivery systems.

Besides CS, cyclodextrin (CD) has become the focus of scientific research in drug delivery systems. A pH-responsive tosyl-*β*-CD-grafted polyethyleneimine (PEI-*β*CD) hydrogels cross-linked by epichlorohydrin (EPI) was successfully prepared [55]. Hydrophobically modified GOD (HmGOD) was successfully immobilized into the hydrogels. The hydrogels possessed glucose-triggered payload release because HmGOD oxidated glucose to gluconic acid, and gluconic acid caused the swelling of the hydrogels, resulting in the drug release.

### 3.2. GOD-Incorporated Microgels

Hydrogels with GOD immobilized or entrapped using as controlled closed-loop delivery platforms have glucose-triggered insulin release profiles. However, insulin release from hydrogel-based systems is delayed due to the slow response rates of hydrogels to changes in glucose concentration. Microgels and hydrogel microparticles based on GOD have highly selective, reversible, and rapid volume phase transitions, depending on the changed glucose concentration fluctuations. The characteristics of glucose-sensitive microgels serve as promising applications in design of self-regulated drug delivery systems [56].

**Figure 8:**
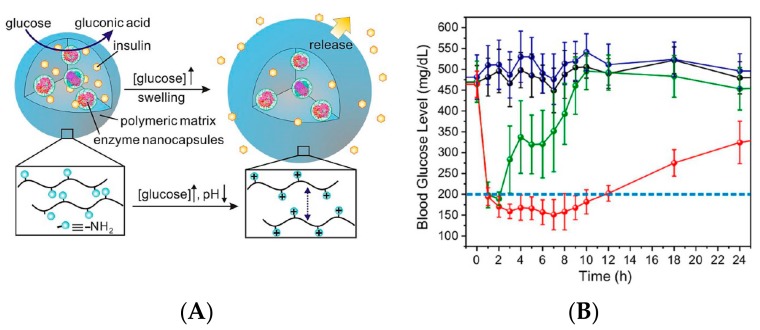
Glucose-sensitive nanocapsule for insulin delivery. (**A**) Schematic of microgel encapsulating insulin and enzyme nanocapsule and glucose-mediated insulin release; (**B**) BGL in diabetic mice after subcutaneous injection with PBS, MGs(E+I), MGs(I), and MGs(E) within 24 h, where MGs(E+I), MGs(I), and MGs(E) were microgels encapsulating insulin and enzymes, insulin alone, enzymes alone, respectively. (Reprinted with permission from [57]).

The injectable microgels, consisting of a physically cross-linked pH-responsive chitosan matrix, GOD- and CAT-containing enzyme nanocapsules, and recombinant human insulin, were fabricated and used for controlled glucose-sensitive insulin delivery [57]. The enzyme nanocapsules were shown in Figure 8A, where enzymes were covalently encapsulated into the nanocapsules, improving enzymatic stability and minimizing enzyme diffusion from the polymer matrix. Continuous swelling led to expansion and dissociation of the polymer network under the enzymatic reactions of GOD and CAT with insulin release under hyperglycemic conditions. The BGL of mice treated by microgels containing insulin with enzyme nanocapsules (MGs(I+E)) were maintained in the normoglycemic state (<200 mg/dL) for 12 h (Figure 8B). However, the BGL of mice injected by microgels containing insulin alone (MGs(I)) quickly declined to a normoglycemic state within 2 h and then steadily increased back to a hyperglycemic state after 2 h. The injectable microgels may find important biomedical applications.

Another kind of microgels integrated with hydrophobically-modified GOD was reported [58]. The microgels were obtained by OA-*g*-ACD copolymer and GOD-CAT dispersion, where the OA-*g*-ACD copolymer was prepared by reacting primary amine of aminated *β*-CD (ACD) with carboxyl of oleic acid (OA). The introduction of OA improved the hydrophobic cavity, thereby increased the encapsulation of insulin. Hyaluronic acid (HA) was also used for preparation of the glucose-sensitive microgels based on GOD [59]. GOD-based microgels with a chemically-crosslinked network of poly(*N*-isopropylacrylamide) (PNIPAM) possessed a fast reversible time response to the changed glucose concentration, while nearly unchanged in size upon adding other saccharides or polysaccharides/glycoproteins [60].

**Figure 9:**
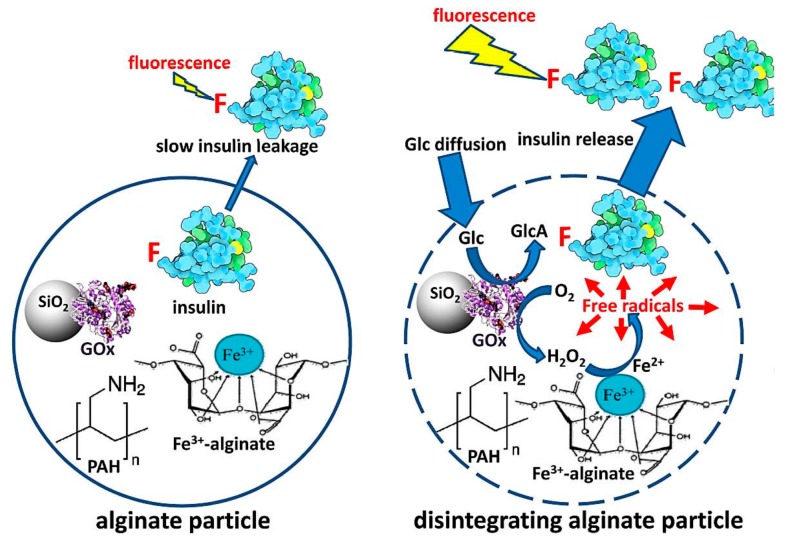
Schematics of alginate particle composition (**left** panel) and reduction in cross-linking (**right** panel) in the presence of glucose with FITC-insulin release. FITC labels are denoted by F. (Reprinted with permission from [61]).

Alginate hydrogel particles cross-linked by Fe^3+^ were designed [61]. GOD was immobilized on amino-functionalized silica nanoparticles (SiO_2_-NPs), producing GOD-SiO_2_-NPs with 15 GOD molecules per SiO_2_-NP. Then a mixture of alginate/FITC-insulin/GOD-SiO_2_-NPs was dropped onto the solution containing FeCl_3_, where FITC-insulin was fluorescein isothiocyanate (FITC)-labeled insulin. The resulted alginate particles were obtained with ca. 4 × 10^8^ GOD molecules per particle. The mentioned alginate particles were additionally stabilized by a poly(allylamine hydrochloride) (PAH) layer on the particles. Additionally, the thin layer of PAH on the alginate particles protected the entrapped insulin from leakage. During the glucose oxidation process, H_2_O_2_ was decomposed, yielding free radicals in a Fenton-type reaction catalyzed by iron cations. These free radicals caused the oxidative breakup of alginate, which reduced the density of the alginate hydrogel particles with preloaded insulin release (Figure 9). The work offers a model for designing new glucose-sensitive drug self-regulated delivery systems.

Sequential thiol-ene and tetrazine click reactions were also used to design GOD-functionalized hydrogel microparticles [62]. PEG-tetra norbornene (PEG-NB) and dithiothreitol were emulsified in a bulk phase of dextran and then photopolymerized, obtaining PEG hydrogel microparticles. Tetrazine-functionalized GOD (Tz-GOD) was conjugated to the unreacted norbornene groups in PEG hydrogel microparticles, obtaining GOD-immobilized hydrogel microparticles. GOD enzyme bioactivity incubated in the microparticles was quantified, and the results revealed that the enzyme activity showed a concentration-dependent increase. The GOD-functionalized hydrogel microparticles have promising applications in glucose-sensitive drug delivery and glucose detection.

The pH-sensitive hydrogels and microgels with chemically or physically cross-linked structures have potential applications in glucose-sensitive drug delivery based on GOD. The 3D structures endow the hydrogels and microgels with reversible swelling and shrinking changes accompanied by pulsatile insulin release. Compared with hydrogels, microgels have promising applications for the design of glucose-sensitive drug delivery systems. Insulin release from hydrogels-based systems is delayed because the response rates of hydrogels to fluctuated glucose concentrations are slower than that of microgels. For both hydrogels and microgels, non-cyclic release profiles limit their in vivo application. Additionally, the enzyme and drug loading capacity should be improved. Enzymatic efficiency within the glucose-sensitive hydrogels or microgels is associated with the amount and activity of the enzymes, which is easily deactivated during the preparation and storage of the platforms. In addition, in the glucose oxidization process the produced H_2_O_2_ also affects the bioactivity of enzymes. Co-immobilized CAT and an optimized preparation method are the effective approaches to maintain the activity of enzymes and prolong the glucose sensitivity for long-term release with promoted development of glucose-sensitive hydrogels and microgels.

## 4. GOD-Mediated Hybrid Mesoporous Silica Nanoparticles

Mesoporous silica nanoparticles (MSN) with highly biocompatible and easily surface functionalization have attracted more and more attention for drug delivery [63,64]. The worldwide research interest in drug delivery of MSN is based on the tailored mesoporous structure and high surface area of MSN [65,66]. Guest molecules can be loaded in the channels of MSN where the weak host–guest interaction of MSN with therapeutic agents has a key role on the drug loading properties. Many strategies are used to enhance the host–guest interaction between MSN and guest molecules to improve the drug-loading capacity and controlled drug delivery [67,68]. Mesoporous silica materials integrated with stimuli-responsive polymers or biomolecules have attracted great attention in drug delivery systems [16,69].

Glucose-sensitive enzyme multilayers-coated mesoporous silica particles (EMC-MSP) were reported [14]. The mesoporous silica particles were coated with GOD and CAT multilayer shells on the surface of MSP cross-linked with GA, and EMC-MSP were obtained. The enzymes immobilized in EMC-MSP were still active, and the GOD activity was calculated, which accounted for 66% of that of the free one. The EMC-MSP had obvious glucose-triggered insulin release profiles, with the release rate regulated by the changing glucose concentration. Importantly, the insulin release was monitored in a repeated on/off manner in the alternate presence/absence of glucose. The insulin release properties were regulated by the thickness of enzyme shell. The EMC-MSP drug delivery system combined the advantages of both high-storage capacity and the glucose-triggered insulin release, and had a great potential in applications for diabetes therapy.

**Figure 10:**
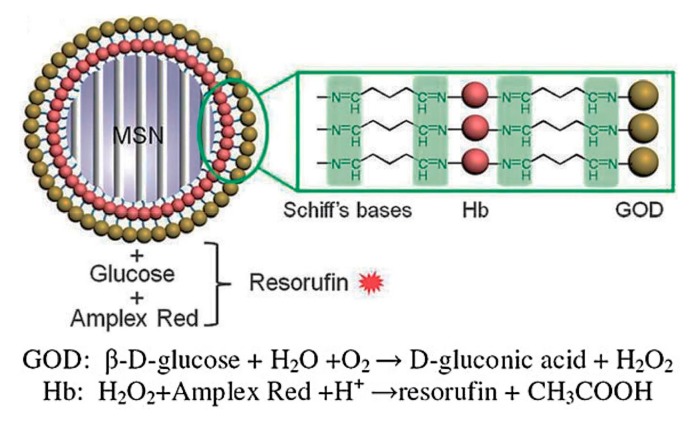
Schematic of hemoglobin (Hb, red) and GOD (blue) immobilized MSN nanoparticles with GA as a cross-linker, and reaction scheme of a coupled enzymatic system catalyzing d-glucose. (Reprinted with permission from [70]).

The glucose-sensitive and autofluorescent protein-coated MSN was fabricated [70]. The covalently-linked hemoglobin (Hb) and GOD multilayers were coated on the surface of MSN by the LbL method (Figure 10). Different protein layer numbers were immobilized on the surface of MSN, obtaining MSN@protein with an average diameter of 100–150 nm. H_2_O_2_ was produced during the glucose oxidization reaction catalyzed by GOD. In the presence of Hb, H_2_O_2_ reacted with the fluorogenic reagent Amplex Red to produce the fluorescent compound resorufin. These unique features make this nanocomposite a good candidate as a cell marker or drug carrier.

An organic–inorganic composite microcapsule was prepared via a combination of LbL self-assembly and biomimetic mineralization [71]. The microcapsules made of sodium polystyrenesulfonate (PSS), poly(dimethyl diallyl ammonium chloride) (PDADMAC), and biomimetic silica denoted by (PSS/PDADMAC)_2_-SiO_2_. The characteristics of the GOD encapsulated in (PSS/PDADMAC)_2_-SiO_2_, such as pH and thermal stabilization, and Michaelis constant, were studied. Compared with free GOD, GOD encapsulated in (PSS/PDADMAC)_2_-SiO_2_ was better at enhancing enzyme stability against changes in temperature and pH, and during long-term storage endowing the hybrid microcapsules with potential applications for the glucose-sensitive drug delivery.

In recent years, gated materials have attracted the attention of scientists and research of using enzymes as functional gating elements has been reported. Using mesoporous silica materials MCM-41, Chen and coworkers prepared the GOD-gated MSN for glucose-sensitive drug delivery [72]. In this system, d-(+)-glucosamine, an effective inhibitor of GOD, was modified on the external surface of MCM-41, obtaining MSN-anchor. Rhodamine B (RB) was loaded into the pores of the MSN-anchor, resulting in MSN-anchor-RB. The MSN-anchor-RB was capped by GOD due to the reaction of GOD and the d-(+)-glucosamine anchored outside the pores of nanocontainers, and the target product (MSN-anchor-RB)@GOD was obtained. Glucose triggered the release of RB from (MSN-anchor-RB)@GOD with higher selectivity. The GOD-gated MSN has potential applications in self-regulated drug delivery systems.

**Figure 11:**
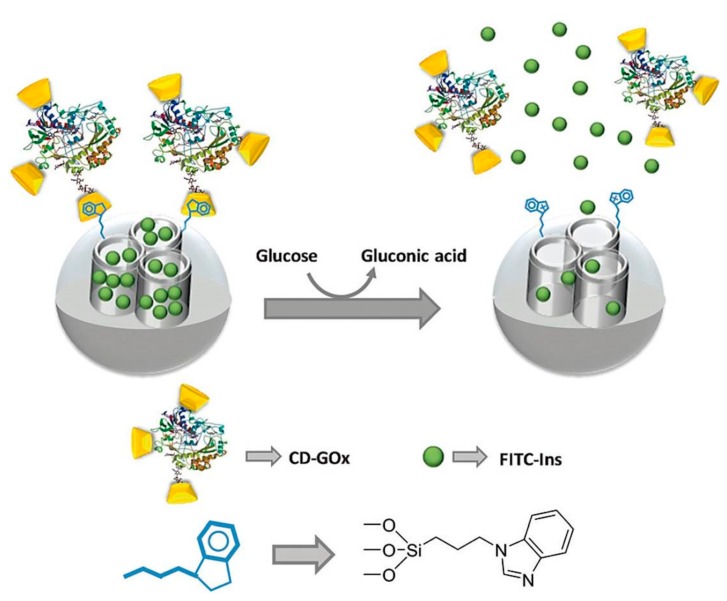
Schematic illustration of glucose-mediated drug release from mesoporous silica nanoparticle capped by *β*-CD-modified glucose oxidase. (Reprinted with permission from [74]).

In addition, a novel gated mesoporous silica nanodevice with *β*-CD-modified GOD (CD-GOD) as the capping agent was reported [73]. The MSN incorporating propylbenzimidazole units was capped with CD-GOD through the formation of inclusion complexes between *β*-CD and the propylbenzimidazole group. The benzimidazole group could protonate by gluconic acid, which induced the uncapping of CD-GOD from the MSN with subsequent preloaded dye release. To further confirm the promising application in glucose-sensitive drug delivery, the authors studied the glucose-triggered FITC-insulin release profiles from the gated mesoporous silica nanodevice [74]. The cap CD-GOD was dissociated from the MSN, and FITC-insulin was released with maintaining insulin activity (Figure 11). A relatively low amount of the drug-loaded nanocontainer released suitable amounts of insulin, which was necessary to decrease the BGL to the regular state. Besides, the system was highly selective. Insulin was delivered only induced by glucose, whereas other saccharides were unable to trigger the insulin release.

**Figure 12:**
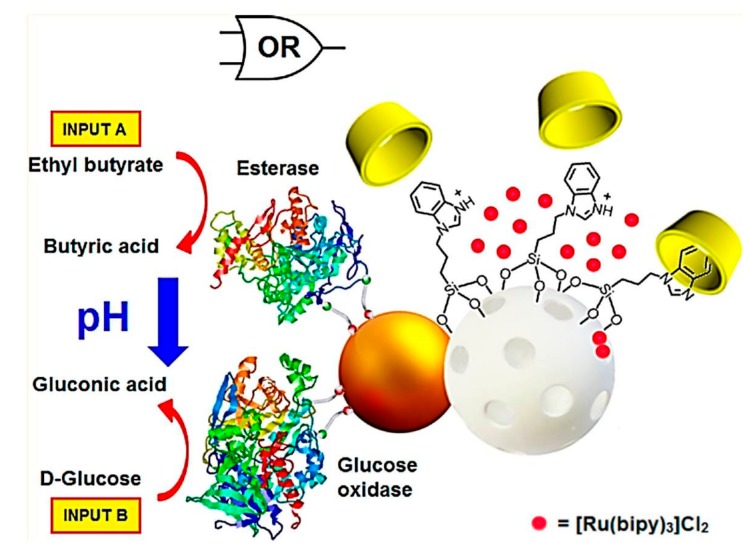
Schematic illustration of Janus-based nanodevice as a smart delivery system controlled by integrated enzymes. (Reprinted with permission from [13]).

Furthermore, the cargo delivery from the gated MSN regulated by an integrated enzyme-based “control unit” was explored [13]. The drug delivery system consisted of Janus-type nanoparticles, which had opposing Au and mesoporous silica faces. The Au side acting as the “control unit” was functionalized with two effectors, i.e., GOD and esterase. The mesoporous silica faces were functionalized with a pH-responsive *β*-CD-based supramolecular nanovalve. GOD oxidized the glucose to gluconic acid, and the esterase hydrolyzed ethyl butyrate to butyric acid. Two kinds of the produced acids led to the reduction of the pH and consequently the opening of the *β*-CD-gated nanovalves with drug release (Figure 12). The Janus-based nanodevice opens a wide range of new possibilities for the development of novel smart delivery systems controlled by enzymes.

Biocompatible MSN has emerged as a promising candidate for drug delivery due to the tunable particle size, pore structure, and surface modification. MSN integrated with GOD have attracted great attention for their applications in glucose-sensitive drug delivery systems. Many strategies are used to enhance the glucose-sensitivity of GOD-based MSN, such as using multilayer and gated materials on the MSN to maintain the payload in the pore of MSN and get “on–off” drug release profiles. However, the entrapment and bioactivity of GOD restrict the development of GOD-mediated hybrid MSN. An ingenious structure design combined with convenient preparation methods will promote the repeated pulsatile drug release profiles of GOD-based MSN, and further promote the promising applications of GOD-based hybrid platforms for diabetes therapy.

## 5. GOD-Mediated Microdevices

Glucose-sensitive drug delivery platforms, such as self-assembly films and polymer vesicles, cross-linking hydrogels and microgels, and MSN have good glucose sensitivity. However, the clinical applications for diabetes therapy of these platforms are limited due to the weak mechanical strength, inaccurate amount of released insulin, low insulin loading, and so on. Microdevices integrated with insulin reservoirs with membranes immobilizing the glucose sensor GOD have attracted great attention. Large reservoir volume offers suitable insulin loading in the microdevices with high and accurate drug loading capacity. Glucose sensor GOD can be immobilized or entrapped in functionalized membranes, which are bounded on the insulin reservoir to prevent or promote insulin release from microdevices in normoglycemic or hyperglycemic conditions.

**Figure 13:**
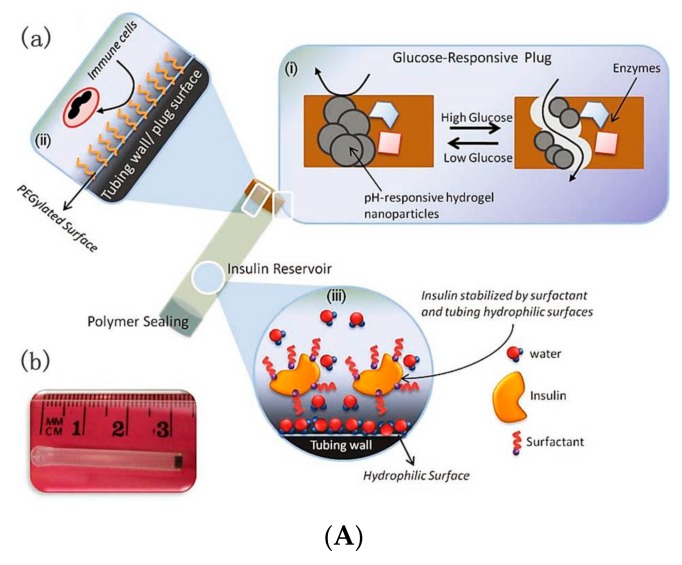

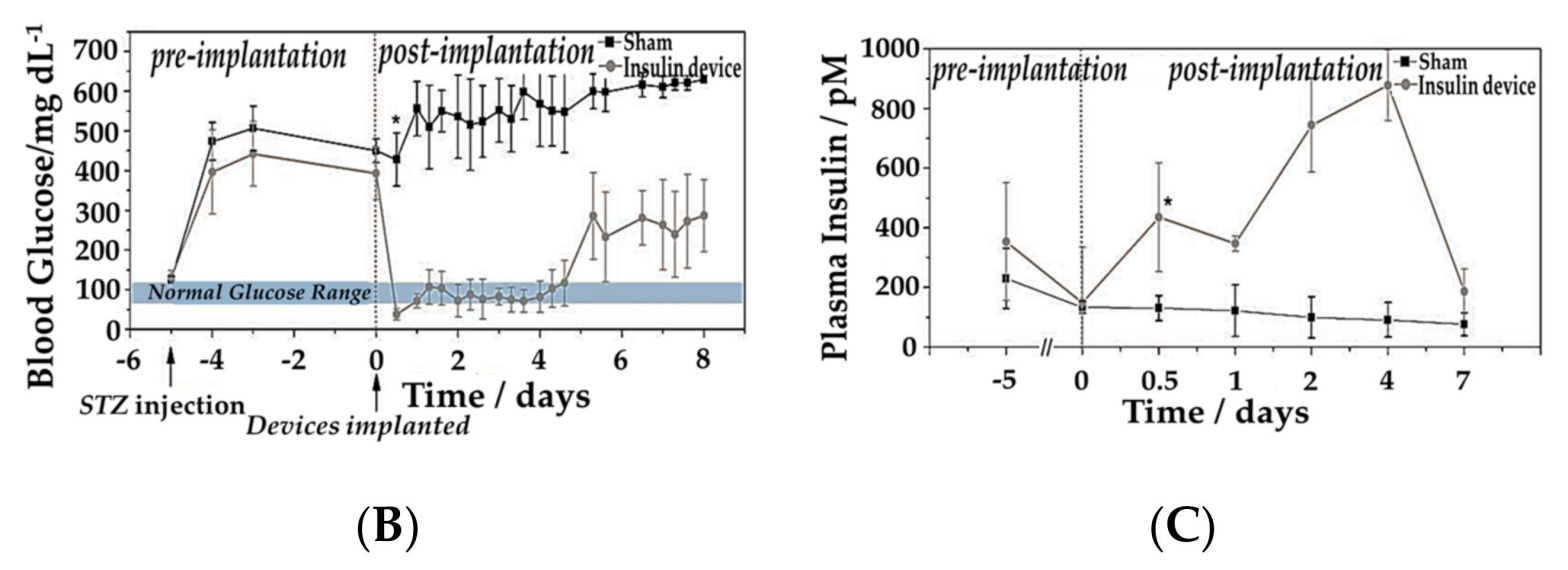
An insulin delivery prototype device for BGL regulation in STZ-induced diabetic rats. (**A**) (**a**) Schematics of an insulin delivery prototype device and its components, (**b**) photograph of insulin delivery device prototype; (**B**) Change in blood glucose after intraperitoneal implantation with saline-filled devices (Sham) or insulin-filled devices (Insulin Device); (**C**) Change in plasma insulin level after intraperitoneal implantation with Sham or Insulin Device. (Reprinted with permission from [75]).

An implantable closed-loop insulin delivery device was designed, which consisted of a multifunctional bio-inorganic nanocomposite glucose-sensitive membrane and insulin reservoir [75]. Using silicone tubing as the insulin reservoir, one end of the tubing was sealed with polymer and the other was integrated with the glucose-responsive plug. The glucose-responsive plug was prepared by cross-linking a mixture of BSA, GOD, CAT, MnO_2_ and pH-responsive hydrogel nanoparticles (poly(*N*-isopropyl acrylamide-*co*-methacrylic acid) nanoparticles) with glutaraldehyde. At hyperglycemic glucose levels, produced gluconic acid enlarged the porosity of the pH-responsive hydrogel nanoparticles with insulin release (Figure 13Aa). The device was modified by PEGylated surface to improve the safety and biocompatibility. In addition, the device was small in favor of the implanted and explanted subcutaneously or intraperitoneally in rats (Figure 13Ab). Insulin was loaded in the device through a syringe with a thin needle. Typical pulsatile insulin release profiles endowed the device with excellent hypoglycemic effect. As shown in Figure 13B, after intraperitoneal implantation, the insulin-filled device had long-term dramatic hypoglycemic effect compared to the sham-filled device, which controlled the blood concentration at normal levels for five days. The reason was that there was an increase in plasma insulin levels, which was associated with the hypoglycemic effect (Figure 13C). The research results endow the implantable closed-loop insulin delivery device with promising application in diabetes therapy.

**Figure 14:**
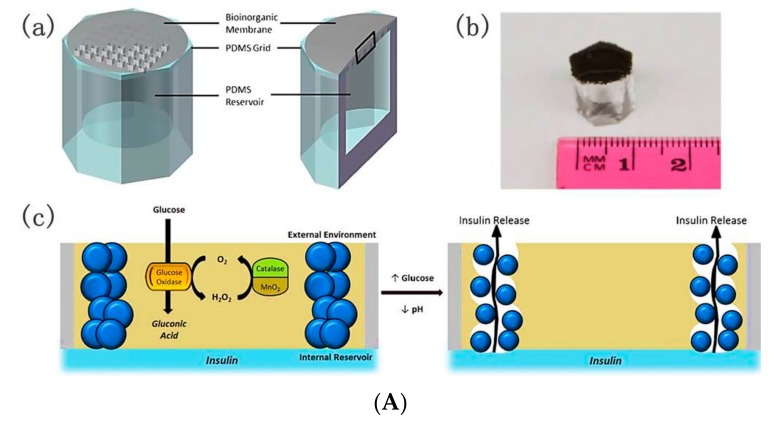

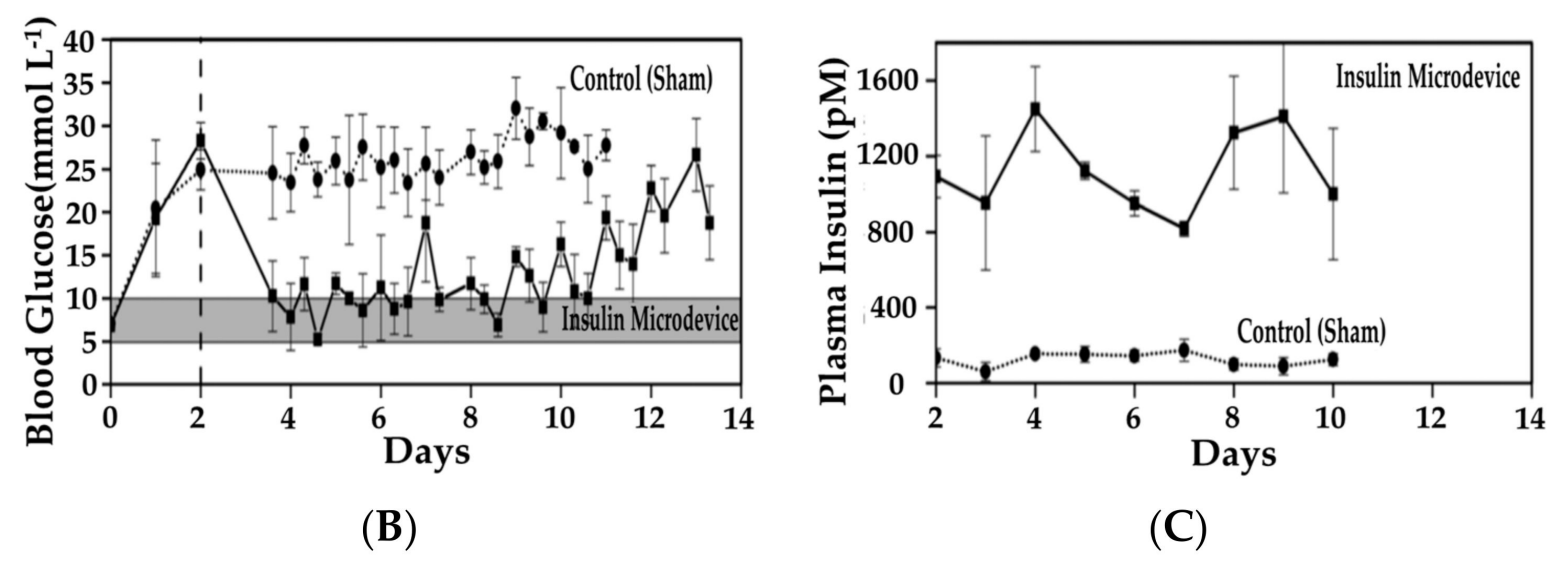
PDMS grid-gel microdevice implanted in STZ-induced diabetic rats. (**A**) (**a**) Schematic representation of PDMS grid-gel microdevice with integrated bioinorganic membrane (with inset for (**c**)); (**b**) Size comparison of the PDMS grid-gel microdevice; (**c**) Cross-sectional diagram and schematic of glucose-triggered insulin release; (**B**) Long-term plasma blood glucose regulations and (**C**) long-term plasma insulin measurements in STZ-induced diabetic rats treated with a microdevice filled with insulin or saline (control) (*n* = 5). Shaded area indicates normoglycemic range. Implantation of microdevices occurred at day 2. (Reprinted with permission from [76]).

Although the previous tubing-shaped microdevices were proven effective in lowering hyperglycemia, the complex fabrication processes and the smaller reservoir volume for insulin storage restrict the applications in drug delivery. A glucose-responsive implantable grid-gel microdevice with a larger insulin reservoir volume was developed [76]. The microdevices consisted of polydimethylsiloxane (PDMS) reservoirs, PDMS grids, and bioinorganic membranes (Figure 14A). The PDMS reservoirs were prepared by bonding a PDMS sheet with insulin reservoir, and the PDMS grids were bonded with bioinorganic membrane. In the bioinorganic membrane there were MnO_2_ nanoparticles, albumin, GOD, CAT, and poly(N-isopropylacrylamide-co-methacrylic acid) (P(NIPAM-*co*-MAA)) hydrogel nanoparticles. PEG was introduced on the PDMS surface to enhance the hydrophilicity of the microdevices surface and improve insulin compatibility with the microdevices. Insulin solution was injected into the PDMS microdevices, resulting in insulin-filled microdevices. In glucose solution, GOD converted glucose to gluconic acid, which produced undesired H_2_O_2_. The MnO_2_ nanoparticles and CAT decomposed harmful H_2_O_2_ and recovered consumed oxygen. Gluconic acid induced the shrinking of the hydrogel nanoparticles with enhanced porosity of the membrane, and as a result, insulin was released from the microdevice reservoir, triggered by glucose (Figure 14Ac). The significant pulsatile glucose-triggered insulin release endowed the device with promising application for diabetes therapy. STZ-diabetic rats implanted with a single microdevice filled with insulin formulation and saline were the insulin microdevice-treated group and the control group, respectively. For the control group, glucose concentrations reached above 20 mmol L^−1^. However, for the insulin microdevice group, the glucose levels were maintained at normoglycemia for at least seven days. Plasma insulin measurements in STZ-diabetic rats confirmed the BGL changes. In the control group, the insulin levels were much lower than normal physiological levels. In the treated group, insulin level increased rapidly after the insulin-filled microdevice implanted with insulin, and were relatively stable over 10 days, demonstrating the hypoglycemic effect (Figure 14B,C). This work promotes the development of self-regulated drug delivery systems.

Glucose-sensitive GOD-based microdevices used for closed loop insulin delivery have real time on-demand drug release profiles and regulate BGL within normoglycemia for a long term. These microdevices consist of insulin reservoirs used for insulin loading, and membranes used for glucose sensor and controlling insulin release. The GOD-based microdevices have excellent mechanical strength compared to other platforms, which offers convenient applications for the regulation of BGL. The insulin reservoir offers high and accurate insulin-loading capacity, which was easily used for drug refilling. In the membrane, there are GOD, CAT, and pH-sensitive polymers. The membranes integrated with insulin reservoirs play an important role in glucose-triggered drug release. How to reinforce the membrane permeability and maintain membrane integrity are factors to consider for the design of GOD-based microdevices. The bioactivity of GOD plays a key role in the long-term, lowering hyperglycemia. Taking into account shape, structure, biocompatibility, and so on, to design intelligent microdevices will promote their clinical applications for diabetes treatment.

## 6. Conclusions

We have reviewed recent developments in the GOD-based glucose-sensitive insulin delivery systems. The self-regulated drug delivery systems can release payload automatically and continuously depending on the elevated BGL. The glucose-sensitive platforms based on GOD have become one of the focuses of diabetes therapy researches, and a series of advances has been made.

LbL films can be easily prepared with controlled membrane thickness and permeability. Even though LbL films cannot provide effective closed-loop insulin release due to the weak mechanical strength, GOD-immobilized or entrapped films is one potential platform for glucose-sensitive drug delivery. Polymer vesicles provide larger volume for insulin loading, however the structure is unable, which depends on the preparation method. Cross-linking hydrogel and microgel provide repeated on–off drug release due to the reversible swelling and shrinking changes. GOD-mediated hybrid MSN integrated with multilayer and gated materials on the MSN provide pulsed drug release. Even though the studies of GOD-based MSN are only in the stage of in vitro drug release, this platform is one potential carrier for glucose-sensitive drug delivery. GOD-mediated microdevices with accurate insulin-loading capacity are a potential candidate for diabetes therapy. The real time on-demand insulin release profiles endow the microdevices with long-term regulation of BGL within normoglycemia. All of these GOD-mediated platforms have promising applications for diabetes therapy.

Even though the progress of GOD-based platforms in glucose-sensitive drug delivery is considerable, some challenges and limitations still restrict the clinical applications in diabetes therapy. Firstly, the bioactivity of GOD during long-term glucose-triggered insulin delivery must be preserved. The bioactivity maintenance of GOD has a key role in the glucose-sensitive drug delivery. During the preparation and storage of GOD-based platforms, the bioactivity of GOD decreased. Adopting simplified preparation of platforms is conducive to maintain the bioactivity of GOD. Secondly, the glucose sensitivities of platforms decrease during the oxidation of GOD, due to the production of H_2_O_2_ and the consummation of dissolved O_2_. Many strategies, such as the combination of GOD with CAT or Hb, using H_2_O_2_-sensitive, hypoxia-sensitive, or hypoxia and H_2_O_2_ dual-sensitive polymers, assist glucose sensitivity. Thirdly, biocompatibility without long-term side effects of the platforms must be considered for the design of GOD-based self-regulated drug delivery systems. For in vivo applications, the platforms must be non-toxic and friendly to the body, and the foreign platforms must not induce inflammation. Many biodegradable and biocompatible materials, such as poly(acrylic acids) and polysaccharides, are adopted in the fabrications of GOD-based matrices. In addition, PEGylation is an effective method to enhance the biocompatibility. Fourthly, the precise dosage of insulin release from glucose-sensitive carriers limits the practical applications. Proper amounts of insulin should be released depending on BGL, that is, rapid and numerous release in response to hyperglycemic state and basal insulin rates at normoglycemic level. Lastly, bioactivity of released insulin is another challenge for glucose-sensitive drug delivery systems. During preparation of the insulin-loaded matrices and the insulin release process, the original bioactivity of insulin must be maintained, which is further used to regulate BGL under hyperglycemic state. Although great efforts are still needed to address some issues, the GOD-based glucose-sensitive drug delivery systems have great potential for the applications in diabetes treatment.

## Figures and Tables

**Table 1:** GOD-mediated platforms.

Method	Platform	Drug Delivery Mechanism
Self-assembly	LbL films	Glucose-induced decomposition of films with insulin permeation
	Vesicles	Dissociation or destruction of vesicles induced by gluconic acid, H_2_O_2_, and hypoxia
Cross-linking	Hydrogels	Structural changes in response to pH changes of microenvironments, or acidic biodegradation of pH-sensitive materials
	Microgels	
Weak physical interaction	Mesoporous silica materials	Permeation changes of multilayers coated on mesoporous silica materials, or open of pores on mesoporous silica materials due to the glucose-induced uncapping of gated materials
Fabrication	Devices with an insulin reservoir	Permeation changes of membrane used for sealing of insulin reservoir
